# Editorial for Special Issue “Molecular Research in Vaccinology and Vaccine Development”

**DOI:** 10.3390/cimb48050533

**Published:** 2026-05-20

**Authors:** Attila Farsang

**Affiliations:** Doctoral School, University of Veterinary Sciences, H-1078 Budapest, Hungary; farsang@gmail.com

Infectious diseases have always posed a significant threat to both humankind and our livestock. Nowadays, we may experience even greater microbial pressures due to globalism, long-haul flights, and environmental effects. New viruses, with new sero- and genotypes are emerging. Without claiming to be exhaustive, just think of Zika virus, COVID-19, SARS, and new genotypes of Infectious Bronchitis Virus (IBV) in poultry or Porcine Reproduction and Respiratory Virus in swine. Due to climate change, new viruses are crossing geographical limits and emerging in new continents and countries where the given disease or pathogen was previously unknown. We can see this in the case of West Nile Virus or Chikungunya virus. These changes and the emerging pathogens involved are posing a constituent challenge for vaccinology and vaccine development. It is in the interest of public and veterinary health to keep pace via the development of state-of-the-art, potent, safe and efficacious vaccines against these diseases. In line with the “One Health” approach (https://www.who.int/health-topics/one-health#tab=tab_1, accessed on 29 April 2026), these microbial challenges are presently being dealt with based on a global view; this perspective acknowledges the significance of zoonotic diseases and recognises what veterinary/human diseases and pathogens are not two separate realms of vaccinology and vaccine development—rather, they form a complex unity with many synergies, interactions and connections.

Another important aspect of this field, which highlights the utmost significance of scientifically sound data and meticulous research is the fact: we must combat the growing anti-vaccination movements with the deployment of safe, efficient and potent vaccines. The research community’s responsibility is to communicate the ways in which deep analysis, scientific efforts and strict regulatory rules govern the market and the authorization of vaccines.

In this context, I am proud to present this Special Issue: “Molecular Research in Vaccinology and Vaccine Development”. This Special Issue is a collection of five original research papers showing state-of-the-art results from studies on vaccinology and vaccine development against various agents (Mycobacterium bovis, Porcine Reproductive, Respiratory Syndrome Virus, SARS-CoV-2, Henipavirus and Dengue virus). The authors come from three countries (Brazil, China and Jordan), reflecting the truly global and collaborative nature of contemporary research in vaccinology.

Liu et. al. [1] focused on Nipah and Hendra viruses, which are lethal zoonotic pathogens with no approved vaccines or therapeutics. In their paper, the authors identified an optimal combination for expressing a Henipavirus-neutralizing antibody 1 × 10^5^ via systematically screened natural and artificial untranslated regions (UTRs). Their findings provide a therapeutic strategy for Henipaviral infections and a blueprint for the development of mRNA-based antibodies against emerging viruses.

Guo et. al. [2] carried out research on Dengue virus, a pathogen that is responsible over 5 million confirmed cases globally, with more than 5000 recorded Dengue-related fatalities [3,4].

Dengue virus (DENV) is a major public health threat in the tropical and subtropical regions of the world. Climate change, a result of global warming, is further expanding DENV-endemic areas, adversely affecting public life and health, especially as no specific drug is available for treating DENV. Vaccines and neutralizing antibodies might be potential tools to fight to DENV pathogenic infections; however, development of a Dengue vaccine is a highly challenging because of antibody-dependent enhancement (ADE). No vaccine against Dengue has yet been developed that can be used in all age groups of severely affected patients. Guo et. al. [2] established a novel method for evaluating antibodies.

The plaque-reduction neutralization test (PRNT) has traditionally been regarded as the gold standard for assessing antibodies against DENV-neutralization capacity; however, it has many shortcomings. For instance, it is labour-intensive and time-consuming [5,6,7]. The authors developed a novel fluorescence-reduction neutralization test (FRNT) for evaluating the neutralizing activity of antibodies against DENV. This method allows for direct assessment of the degree of viral infection in host cells based on fluorescence intensity at specific wavelengths, which correlates to the neutralization potency of the antibody, and allows practitioners to calculate the neutralizing capacity of the antibody; additionally, it offers enhanced speed, efficiency, and objectivity.

It is believed that the innovative FRNT could provide substantial support for the development of anti-DENV vaccines and antibodies, with potential applications extending toother flaviviruses such as Zika-virus (ZIKV), West Nile Virus (WNV), or Japanese Encephalitis Virus (JEV).

Souan et. al. [8] studied SARS-CoV-2, the trigger of the COVID-19 pandemic [9]; This pandemic was an unparalleled international health crisis, profoundly affecting society, economics, and healthcare systems all around the globe [10]. Throughout the pandemic, healthcare workers (HCWs)—including doctors, nurses, paramedics, and support personnel—have faced unprecedented challenges. This study gave a vital insight into the effectiveness of vaccination, the durability of immunity, and the consequences of occupational health and public health measures by understanding the complexities of these immune responses. This is the first time when it could have been demonstrated that HCWs maintain both cellular and humoral immune responses against the SARS-CoV2 virus for more than ten months following immunization.

Wang et. al. carried out research on Porcine Reproductive and Respiratory Syndrome (PRRS) [11]. PRRS is a highly contagious infection that affects domestic pigs of all ages. It is characterized by reproductive failure in sows and respiratory distress in growing pigs, posing a significant danger to the pork industry on a global scale [12,13]. Due to the extensive genetic and antigenic variation in PRRSV, as well as its rapid mutability and evolution, a variety of emerging and re-emerging virus strains have continued the cause epidemics worldwide [14,15]. Therefore, PRRS prevention and control can be challenging, although several PRRSV vaccines have been developed. Scientists and veterinarians are currently exploring new strategies for vaccine design. An ideal vaccine against PRRSV should rapidly induce a high level of NAbs, as well as a specific cellular response against the virus. The presence of specific antibodies and traces of PRRSV in the serum suggests that the early antibody response does not provide protection against PRRSV infection [16,17,18,19]. The authors developed an assay to monitor neutralizing antibodies with increased sensitivity. This assay may serve as a new platform to evaluate next-generation PRRS vaccines.

Last, but not least, Pagani et. al. [20] carried out excellent research on toxin–antitoxin system highlighting the Impact of Genomic Deletion RD16 on the Expression of the Mycobacterium bovis BCG Moreau VapBC47. Mycobacterium bovis BCG is the only available vaccine that can be used against tuberculosis. However, most studies on the VapBC family are focused on the structure and function of VapC toxins and their interactions with cognate VapB antitoxins [21,22,23,24,25]. The authors showed that rv3407-rv3408 comprise an operon, the expression of which is increased in BCG Moreau because of the RD16 deletion. As previously shown, this deletion leads to rv3406 constitutive expression in Moreau; here, we corroborate that this mutation has a wider polar effect than previously detected, which also interferes in the transcription of rv3407 (vapB47) and rv3408 (vapC47). Their studies shed light on the regulation of VapBC systems and on the impact of the BCG Moreau RD16 deletion in the expression of adjacent genes, contributing to a better understanding of the physiology of BCG Moreau.
